# Validation of a tri-axial accelerometer for measuring physical activity in patients with subacute stroke

**DOI:** 10.3389/fresc.2024.1496515

**Published:** 2025-01-09

**Authors:** Yosuke Kimura, Yoshiki Suzuki, Hiroki Kubo, Keishi Yoshida, Tomohiro Ota, Natsuki Shimizu, Masashi Kanai

**Affiliations:** ^1^ Department of Biomedical Engineering, Faculty of Life Sciences, Toyo University, Saitama, Japan; ^2^ J-SPURT: Japanese Stroke & Physical Activity Multiple Center Research Team, Saitama, Japan; ^3^ Tokyo Shinjuku Medical Center, Department of Rehabilitation, Japan Community Healthcare Organization, Tokyo, Japan; ^4^ Department of Physical Therapy, Faculty of Nursing and Rehabilitation, Konan Women’s University, Hyogo, Japan; ^5^ Department of Rehabilitation, Senri-Chuo Hospital, Osaka, Japan; ^6^ Department of Rehabilitation and Care, Hatsudai Rehabilitation Hospital, Tokyo, Japan; ^7^ Department of Physical Therapy, Faculty of Health and Medical Care, Saitama Medical University, Saitama, Japan; ^8^ Institute of Transdisciplinary Sciences for Innovation, Kanazawa University, Ishikawa, Japan

**Keywords:** stroke, physical activity, energy expenditure, accelerometer, validity

## Abstract

**Purpose:**

This study aimed to validate the accuracy of the Active Style Pro HJA-750C (ASP) in measuring metabolic equivalents (METs) during walking and reaching tasks in individuals with subacute stroke using a respiratory gas analyzer as a reference.

**Methods:**

Twenty-three hospitalized patients with subacute stroke participated in this study. They performed sitting and standing reaching tasks, as well as walking while wearing a VO2 Master respiratory gas analyzer and ASP devices on both the paretic and non-paretic sides. The METs values recorded by the ASP were compared with those obtained using a VO2 Master respiratory gas analyzer. Pearson's correlation coefficients were calculated for each task, and Bland–Altman plots were used to assess the agreement between the two methods.

**Results:**

The ASP demonstrated good concurrent validity, with correlation coefficients of 0.71 and 0.74 for the sitting reaching task, 0.75 and 0.79 for the standing reaching task, and 0.83 and 0.85 for walking when the ASP was placed on the paretic and non-paretic sides, respectively. Bland–Altman analysis indicated no significant fixed or proportional errors. The ASP accurately measures METs whether worn on the affected or unaffected side of the waist.

**Conclusion:**

The ASP provides valid measurements of physical activity during walking and reaching tasks in patients with subacute stroke. These findings suggest that ASP is a valuable tool for monitoring physical activity in clinical rehabilitation settings.

## 1 Introduction

Stroke is the second leading cause of death worldwide and results in various sequelae, including motor paralysis, balance disorders, and cognitive impairment (1). Consequently, individuals who have experienced stroke often become inactive (2). Previous studies have shown that hospitalized and community-dwelling individuals with stroke spend 70%–80% of their daytime sedentary activities and engage in an average of less than 10 min of moderate-to-vigorous intensity physical activity (3–5). This inactive lifestyle is associated with various adverse health outcomes, including depression, stroke recurrence, and mortality (6–8). Conversely, studies consistently revealed that higher levels of physical activity among patients with stroke lead to improved skeletal muscle function and enhanced functional outcomes (4, 9–11). Therefore, promoting physical activity in patients with stroke is important for overall health and functional recovery.

To effectively promote physical activity in patients with stroke, it is important to accurately measure their physical activity levels. Previous studies have employed various methods for this purpose, including behavioral observation, questionnaires, and accelerometers (12). Among these, accelerometers are a non-invasive and convenient option for monitoring physical activity in clinical settings. However, studies on the validity and reliability of different accelerometers have yielded inconsistent results (12–16). These inconsistencies may arise from variations in algorithms and device performance, such as whether the devices operate on one or three axes and their resolutions. Additionally, the specific movement patterns of individuals with stroke, such as asymmetric walking and the varying severity of impairments, may also contribute to the inconsistency observed in previous studies. Therefore, the usefulness of accelerometers in patients with stroke must be verified by considering both device properties and the characteristics of the participants.

The Active Style Pro HJA-750C (ASP) may be a suitable accelerometer for measuring physical activity in patients with stroke. The ASP is a relatively inexpensive device with a 3-axis accelerometer, offering good resolution and dynamic range, which allows it to capture both rough and subtle movements across the three axes (17–19). Previous studies have reported that the ASP estimates in metabolic equivalents (METs) with high accuracy in healthy adults and has been widely used for community-dwelling older adults and patients with stroke in clinical settings (4, 6, 8, 9, 11, 17, 18, 20, 21). However, its validity in patients with stroke has not been sufficiently investigated.

This study aimed to validate the accuracy of the ASP in measuring physical activity levels during walking and reaching tasks in patients with subacute stroke who can walk independently, using a respiratory gas analyzer as the reference standard. We hypothesized that the ASP would effectively measure physical activity levels during these tasks in patients with subacute stroke.

## 2 Method

### 2.1 Study setting and participants

Patients with subacute stroke were recruited for this study from a 37-bed convalescent inpatient rehabilitation ward between July 2021 and March 2023. Stroke diagnosis was confirmed through clinical examination by a physiatrist and imaging tests (computed tomography or magnetic resonance imaging) conducted by a radiologist. The inclusion criteria were as follows: first stroke affecting the cerebral hemisphere, diagnosis of cerebral hemorrhage or cerebral infarction, ability to walk continuously for 5 min without human assistance, sufficient cognitive ability to understand the tasks in this study protocol and provision of informed consent. The exclusion criteria included comorbid cardiac or respiratory pathologies or decompensated chronic conditions.

We calculated the sample size needed for the correlation analysis of the METs values measured by the ASP and gas analyzer. Assuming a power of 80%, a significance level of *α* = 5%, and a Pearson's correlation coefficient of *r* = 0.6 (13), we estimated that 20 participants would be required.

### 2.2 Experimental protocol

#### 2.2.1 Accelerometer

**Figure 1:**
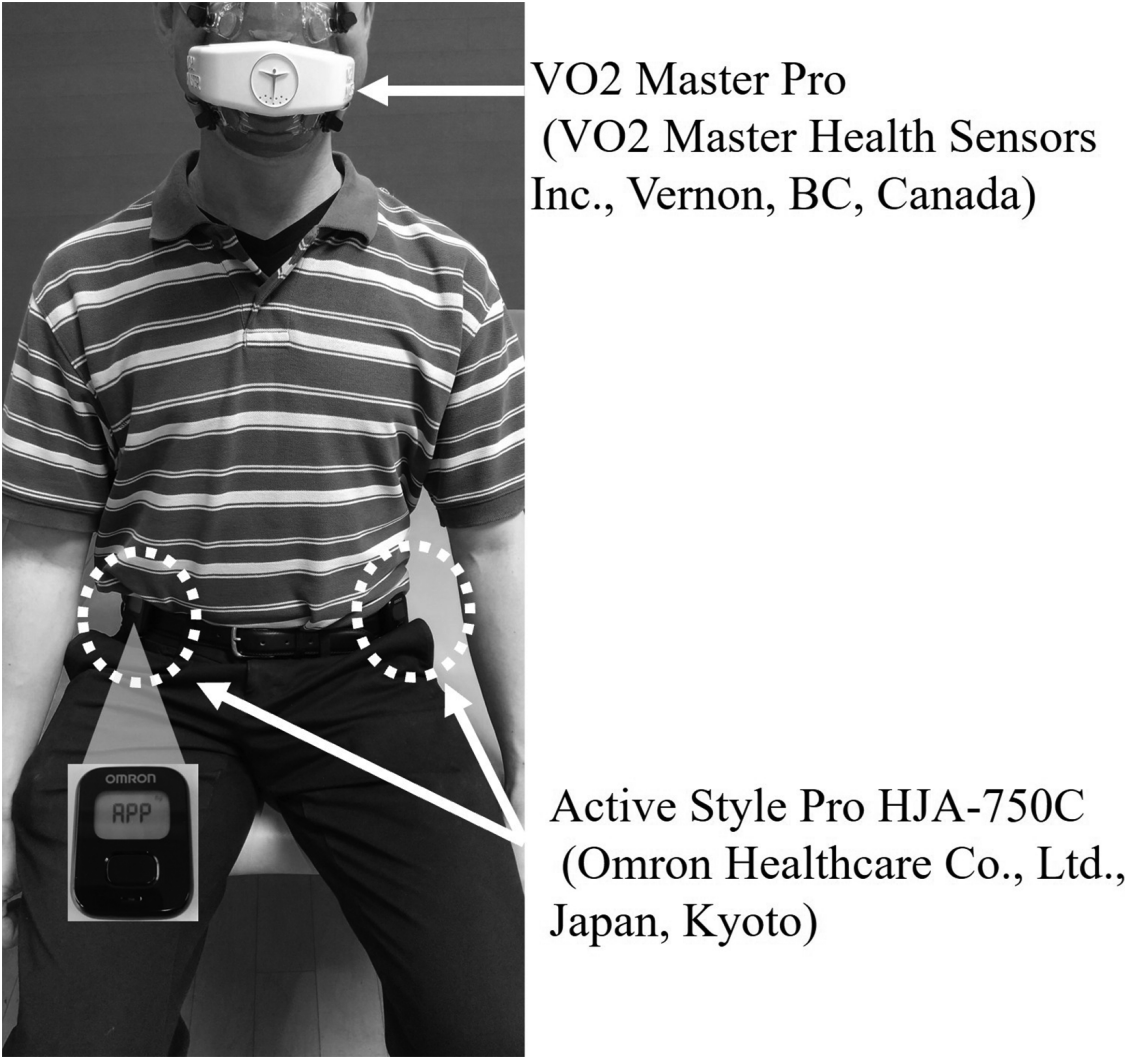
Locations of portable respiratory gas analyzer and accelerometer placement.

The ASP (Omron Healthcare Co., Ltd., Kyoto, Japan; 522 mm × 402 mm × 12 mm; approximately 23 g, including the battery), which is widely used to measure physical activity in daily life among hospitalized and community-dwelling older adults in Japan, was employed in this study. The ASP estimates METs at intervals of either 10 s or 60 s, based on combined accelerations detected by an internal triaxial accelerometer; for this study, a 60-s epoch was selected. The criterion-related validity of MET estimations by the ASP in healthy adults has been validated using the Douglas bag method. The device recorded anteroposterior (*x*-axis), mediolateral (*y*-axis), and vertical (*z*-axis) accelerations with a resolution of 3 mG at a sampling frequency of 32 Hz. Each signal from the triaxial accelerometer underwent high-pass filtering with a cut-off frequency of 0.7 Hz to eliminate the gravitational acceleration component. The device utilizes three distinct equations to calculate activity intensity contingent upon the type of activity, as previously described and validated (17–19, 21). The detailed algorithm used by the ASP to convert raw acceleration data into METs has been thoroughly described in the previous study (19). Participants wore two ASP devices, each placed on their waist belt near the anterior superior iliac spine, with one on the paretic side and the other on the nonparetic side (Figure 1).

#### 2.2.2 Respiratory Gas analyzer

The VO2 Master Pro (VO2 Master Health Sensors Inc., Vernon, BC, Canada) was employed to establish the VO2-derived energy expenditure (EE) and served as the criterion measure against the ASP. The device weighs 0.32 kg and was powered by a single AAA battery. The VO2 Master features a passive pumpless gas sampling system, a galvanic fuel cell O2 sensor, and a differential pressure flow sensor. Upon activation, the VO2 Master was automatically calibrated to ambient air, adjusting for gas concentration, temperature, humidity, and barometric pressure. Prior to each data acquisition session, the portable gas analyzer was calibrated following the manufacturer's guidelines. Once calibration was completed, breath-by-breath analysis of VO2 (L/min) was conducted at 60 s intervals. METs for each movement task measured by the VO2 Master were calculated by dividing the oxygen uptake during the task by the oxygen uptake while sitting quietly. When calculating the METs for each task using the VO2 Master, the oxygen intake during the first 2 min after the start of each task was excluded from the recording to ensure a stable state of oxygen consumption (22). There are no previous studies that have directly tested the validity of the VO2 Master by calculating METs. However, the reliability and criterion-related validity of the VO2 Master using VO2 as the index in healthy adults have been previously validated by comparing the Douglas bag method and stationary exhaled gas analysis equipment (23, 24).

#### 2.2.3 Procedure

The experiments were conducted in the hospital's rehabilitation room. In this study, we employed three motor tasks as test tasks: seated reaching, standing reaching, and gait (Supplementary Figure 2). Participants were instructed to refrain from eating for 3 h before the experiment. The testing protocol was as follows.

▪ Lying on a mat platform table (7 min)
▪ Unsupported sitting on a mat platform table (4 min)
▪ Reaching task while sitting on a mat platform table (4 min)
▪ Rest in a sitting position on a mat platform table
▪ Perform a reaching task while standing (4 min)
▪ Rest in a sitting position on a mat platform table
▪ Walking (5 min)

Sufficient rest time was allotted between each task to ensure that the participants' gas-exchange levels returned to baseline before the execution of the subsequent task.

During the experimental protocol, participants performed the tasks while wearing the two ASPs and the VO2 Master. The detailed procedures for the sitting and standing reaching tasks are based on previous studies (22, 25). For the sitting-reaching task, participants sat on a mat platform table without a backrest and were instructed to use their non-paretic upper limbs to move book clips from a table placed on one side of the body to the other side of the table. In the standing reaching task, participants used the non-paretic upper limb to move book clips from a similarly positioned table, as in the sitting task. The pace of reaching in both positions was set at one reach every 3 s, with a digital auditory metronome used to dictate the timing. For the walking test, participants were instructed to walk back and forth along a 20-m straight track as many times as possible within 5 min using their usual walking mode (i.e., with or without an assistive device) at a self-selected walking speed.

### 2.3 Demographic and clinical variables

The demographic clinical assessment of patients included age, sex, time since stroke onset, type of stroke, body mass index, Motricity Index (MI) (26), Functional Ambulation Categories (FAC) (27), 10 m walking test at a comfortable speed, and the Functional Independence Measure (FIM) (28). The MI evaluates the strength of hip flexion, knee extension, and ankle dorsiflexion on the affected side using methods similar to manual muscle testing. Total scores range from 0 to 100, with higher scores reflecting greater muscle strength. The FAC, an observational scale validated for patients with stroke, was used to categorize gait independence into six categories based on the level of physical support required. The FIM (score range, 18–124) measures basic activities of daily living across 18 items, relating to the degree of independence from assistance.

### 2.4 Statistical analysis

To understand the characteristics of the participants, we calculated descriptive statistics based on the scale properties of each variable and data distribution. Means and standard deviations were used to describe the interval scale variables. Nominal-scale variables were presented as frequencies and percentages.

Pearson's correlation coefficients were used to assess the concurrent validity of the METs values estimated by the ASP and VO2 Master for each movement task. Correlation coefficients between 0.00 and 0.30 were considered negligible, 0.31–0.49 as low, 0.51–0.70 as moderate, 0.71–0.90 as high, and 0.91–1.00 as very high (29). Bland–Altman plots with limits of agreement (LoAs) were used to evaluate the agreement between the measurements for each movement task. Fixed error was assessed by calculating the mean of the differences. Fixed bias was considered present if the 95% confidence interval (95% CI) of the mean difference included 0. Proportional error was assessed by linear regression analysis of the Bland–Altman plot. If the regression was significant, proportional error was considered present.

Statistical analyses were performed using SPSS software (version 24.0; IBM, Tokyo, Japan). The significance level was set at 5% for all analyses.

## 3 Results

Twenty-three patients with subacute stroke participated in this study. The clinical and demographic characteristics of the study participants are presented in Table 1. The participants were predominantly middle-aged to older adults with a slightly higher proportion of males and individuals with cerebral infarctions. More than 60% of the participants can walk independently without supervision (FAC 4 or 5). The mean gait speed is 0.65 (0.38) m/s.

**Table 1:** Characteristics of participants.

Variable	*n* = 23
Age, years	65.2 (14.3)
Sex, Male/Female	15 (65.2%)/8 (44.8%)
Type of stroke, Infarction/hemorrhage	14 (60.9%)/9 (39.1%)
BMI, kg/m^2^	23.6 (4.9)
Time after stroke, days	105.5 (49.1)
Motricity index: Lower limb, points	71.8 (17.9)
FAC, 3/4/5	8 (34.8)/6 (26.1)/9 (39.1)
Gait speed, m/s	0.65 (0.38)
FIM, points	110.0 (11.9)

BMI, body mass index; FAC, functional ambulation category; FIM, functional independence measure.

Values are presented as mean (standard deviation) or number of participants (%).

Table 2 reports MET values and Pearson's correlation coefficients for each movement task measured by the ASP and gas analyzer. Similar results are observed when comparing the MET values of the ASP between the affected and non-affected sides across all movement tasks. The correlation coefficients for sitting and standing reaching tasks are consistently good, while the correlation coefficient for walking is excellent on both the affected and non-affected sides.

**Table 2:** METs value and Pearson's correlation coefficients for each movement task measured by accelerometer and gas analyzer.

	VO2 Master	Affected side		Non-affected side	
		ASP	*r*	ASP	*r*
Reaching in sitting	1.52 (0.15)	1.55 (0.16)	0.71	1.57 (0.17)	0.74
Reaching in standing	1.72 (0.17)	1.77 (0.19)	0.75	1.74 (0.17)	0.79
Walking	3.18 (0.51)	3.20 (0.59)	0.83	3.23 (0.64)	0.85

Values are expressed as mean (standard deviation) and as metabolic equivalents (METs) or Pearson's correlation coefficients.

All p-values for Pearson's correlation analyses were less than 0.001.

Abbreviations: MET, metabolic equivalents; ASP, active style pro HJA-750C.

**Figure 2:**
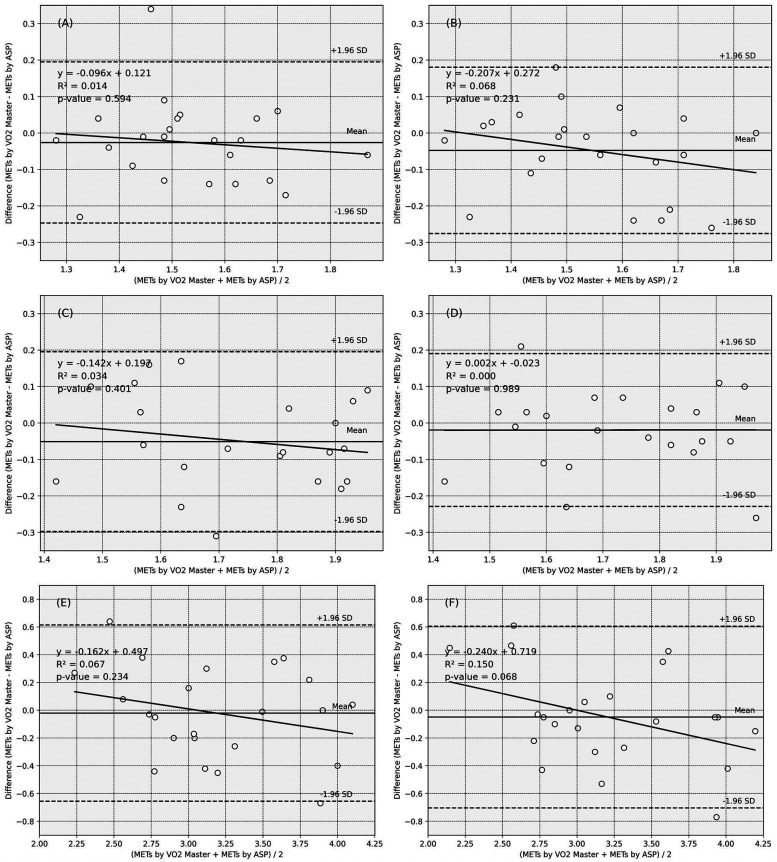
Bland–Altman plots of the ASP_METs and VO2 Master_METs. Solid line: the mean of the difference, dotted line: range of limit of agreement, chain line: 95% CI of the lower and upper limits of agreement. **(A)** Reaching tasks in sitting_affected side, **(B)** Reaching tasks in sitting_non-affected side, **(C)** Reaching tasks in standing_affected side, **(D)** Reaching tasks in standing_non-affected side, **(E)** Walking_affected side, **(F)** Walking_non-affected side.

Figure 2 illustrates the Bland-Altman plots and the limits of agreement. The fixed error is less than approximately −0.05 across all analyses. The plots are generally within the limits of agreement, and visual assessment revealed no evident biases indicative of fixed or proportional errors.

The objective indicators of the fixed and proportional errors are listed in Supplementary Table 1. For all movement tasks, the 95% CI values of the mean differences between the MET values obtained from the ASP and gas analyzer included 0, regardless of whether the ASP was placed on the affected or unaffected side. Additionally, none of the regressions from the Bland–Altman plots are statistically significant.

## 4 Discussion

Our findings revealed that the ASP has high concurrent validity in measuring METs values during walking and reaching tasks in patients with subacute stroke, using the respiratory gas analyzer as the reference standard. The Bland–Altman analysis and mean differences revealed no significant fixed or proportional errors between the ASP and the respiratory gas analyzer. Additionally, the METs values were comparable to those obtained from the respiratory gas analyzer, regardless of whether the ASP was worn on the affected or unaffected side of the participant's waist. These findings suggest that the ASP is a reliable tool for measuring physical activity in patients with subacute stroke who can walk without physical assistance.

Previous studies have reported that some accelerometers fail to accurately measure the energy expenditure of patients with stroke (12–16). Patients with stroke often exhibit various functional impairments such as motor paralysis and hypertonia, leading to diverse movement patterns. For instance, walking may be characterized by slower speed and rough movements due to exaggerated muscle responses and inefficient biomechanics. Similarly, during reaching tasks, reduced postural control limits the range of motion of the center of gravity. As a result, the accuracy of measuring energy expenditure using accelerometers in patients with stroke can produce unreliable results depending on the equipment's performance and underlying algorithm. The ASP has a tri-axis accelerometer with good dynamic range and resolution. In addition, the ASP employs an algorithm that distinguishes between walking and other daily activities to estimate exercise intensity (17–19). These features may contribute to the ASP's relatively accurate measurement of METs during walking and reaching tasks in ambulatory patients with stroke.

Compared to previous studies that evaluated METs during reaching and walking in patients with stroke using a respiratory gas analyzer, the findings of the present study were largely consistent with those reported by Kafri et al. (22) using the COSMED K4b2 system (COSMED, Rome, Italy) for the reaching task in standing (mean, 1.96 [22] vs. 1.72 [This study]) and walking (3.27 [22] vs. 3.18 [This study]). However, when compared to the study by Verschuren et al. (30), which used the METAMAX system (Cortex Medical, Leipzig, Germany), our study revealed higher MET values during the walking task (2.13 (0.56) to 2.84 (0.60) [30] vs. 3.18 (0.51) [This study]). This discrepancy may be due to differences in walking pace; Verschuren et al. (30) measured METs while walking at a comfortable pace, whereas both our study and Kafri et al. (22) instructed participants to walk at their maximum pace. Thus, despite some discrepancies with previous studies due to differences in participant characteristics and measurement protocols, the METs values obtained in this study for reaching and walking exercises were generally comparable and consistent with those reported in previous studies.

This study had certain limitations. First, we only examined the MET values during a few motor tasks. In daily life, various movement activities such as transferring, standing up, and getting up are performed. However, since the respiratory gas analysis was used as the reference, it was necessary to select movement tasks that could be sustained and repeated for a specific duration. Future studies should assess whether the ASP can provide valid measurements for additional movement tasks. Furthermore, given that moderate-to-vigorous physical activity (MVPA) or vigorous physical activity (VPA) was minimal even among relatively healthy, age-matched community-dwelling individuals (31), future research may prioritize investigating physical activities below the MVPA threshold and assessing their validity. Second, this study included participants who could walk without physical assistance and had no comorbidities, such as respiratory or circulatory diseases. The participants also had moderate-to-mild motor paralysis and were mostly middle-aged to young old people. Therefore, the generalizability of these findings to older patients with stroke, those with comorbidities, and severe motor paralysis remains unclear. Third, this study did not evaluate the NIHSS, widely regarded as the gold standard for assessing stroke severity, particularly in the acute phase. Instead, we assessed the FIM and MI, which are critical tools for evaluating functional status and motor impairments in individuals with stroke during the convalescent phase in an inpatient rehabilitation setting. Although these measures do not directly assess stroke severity, they may provide valuable insight into its severity within this population.

## 5 Conclusions

This study revealed that the ASP, equipped with a triaxial accelerometer, can provide a reasonable estimate of METs values for walking and reaching tasks in patients with subacute stroke. Additionally, whether the ASP was worn on the affected or unaffected side of the participant's waist, the METs values were comparable to those obtained using the respiratory gas analyzer. These findings suggest that the ASP may be a valuable tool for managing physical activity in clinical settings for patients with stroke. However, it is important to note that these findings are limited to patients who can walk without physical assistance, are relatively young, and do not have severe comorbidities.

## Data Availability

The raw data supporting the conclusions of this article will be made available by the authors, without undue reservation.
