# Supplementary material for: Validation of a tri-axial accelerometer for measuring physical activity in patients with subacute stroke
Source: Front Rehabil Sci. 2025 Jan 9;5:1496515. doi: 10.3389/fresc.2024.1496515 (PMC11754406; doi:10.3389/fresc.2024.1496515)
Supplement: Supplementary file 1 [file Datasheet1.pdf]

Supplementary Material

1 Supplementary Figure

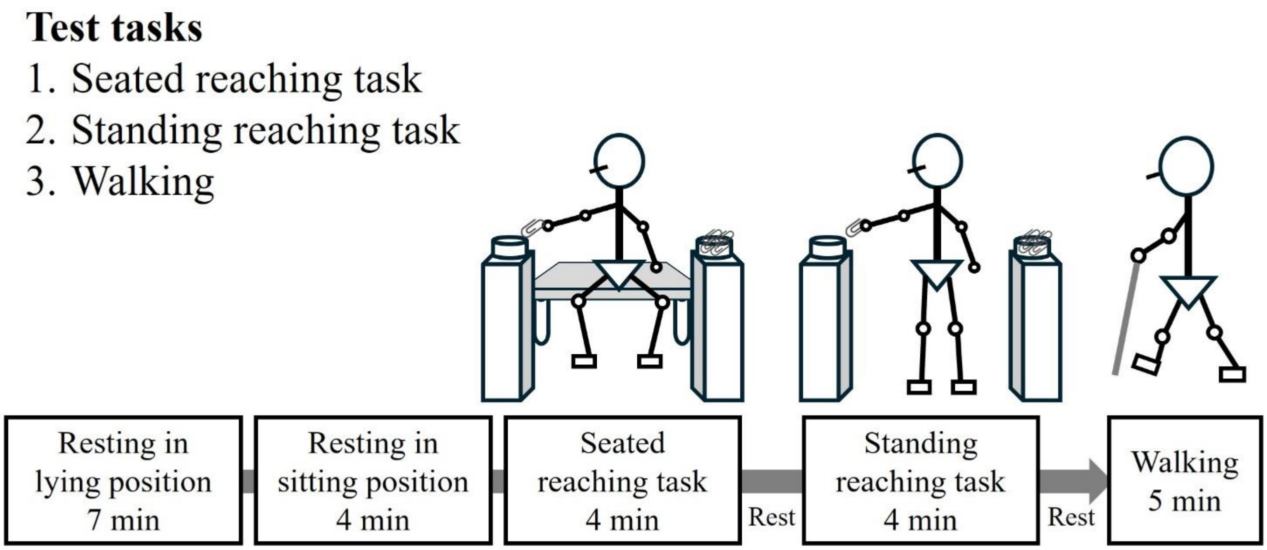

Supplementary Figure 1. Three motor test tasks and experimental protocol

2 Supplementary Table

Supplementary Table1. Results of fixed error, proportional error and LoA.

	Fixed error (95% CI)	Proportional error (r^2) p-value	LoA Lower-upper	95%CI lower	95% CI upper
Reaching in sitting affected side	-0.03 (-0.07 to 0.02)	0.01 0.60	-0.25 to 0.20	0.36 to -0.19	0.13 to 0.30
Reaching in sitting unaffected side	-0.05 (-0.10 to 0.00)	0.07 0.23	-0.28 to 0.19	-0.39 to -0.21	0.12 to 0.30
Reaching in standing affected side	-0.05 (-0.11 to 0.01)	0.03 0.40	-0.30 to 0.20	-0.43 to -0.23	0.13 to 0.32
Reaching in standing unaffected side	-0.02 (-0.01 to 0.03)	0.00 0.99	-0.23 to 0.20	-0.34 to -0.17	0.13 to 0.30
Walking affected side	-0.02 (-0.16 to 0.12)	0.07 0.23	-0.67 to 0.63	-0.99 to -0.48	0.44 to 0.94
Waling unaffected side	-0.05 (-0.20 to 0.10)	0.15 0.07	-0.72 to 0.62	1.04 to -0.52	0.43 to 0.95

Abbreviations: CI, confidential intervals; LoA, limits of agreement.
